# Metabolomic Fingerprint of Behavioral Changes in Response to Full-Spectrum Cannabis Extracts

**DOI:** 10.3389/fphar.2022.831052

**Published:** 2022-01-25

**Authors:** Zaid H. Maayah, Pamela J. F. Raposo, Heidi Silver, Rupasri Mandal, Lee Ellis, Abrar S. Alam, Shingo Takahara, Mourad Ferdaoussi, Kyle E. Mathewson, Dean T. Eurich, Karim Fouad, David S. Wishart, Jason R. B. Dyck

**Affiliations:** ^1^ Cardiovascular Research Centre, Department of Pediatrics, Faculty of Medicine and Dentistry, University of Alberta, Edmonton, AB, Canada; ^2^ Faculty of Rehabilitation Medicine - Physical Therapy, University of Alberta, Edmonton, AB, Canada; ^3^ The Metabolomics Innovation Centre (TMIC), University of Alberta, Edmonton, AB, Canada; ^4^ Department of Biological Sciences, University of Alberta, Edmonton, AB, Canada; ^5^ National Research Council of Canada, Halifax, NS, Canada; ^6^ Neuroscience and Mental Health Institute, Faculty of Medicine and Dentistry, University of Alberta, Edmonton, AB, Canada; ^7^ Department of Psychology, Faculty of Science, University of Alberta, Edmonton, AB, Canada; ^8^ School of Public Health, University of Alberta, Edmonton, AB, Canada

**Keywords:** cannabis, metabolomics, behavior, THC - tetrahydrocannabinol, CBD - cannabidiol

## Abstract

Numerous existing full-spectrum cannabis extract products have been used in clinical trials for the treatment of various diseases. Despite their efficacy, the clinical use of some of these full-spectrum cannabis extracts is limited by behavioral side effects such as cognitive dysfunction and impaired motor skills. To better understand what constitutes cannabis-induced behavioral effects, our objective was to identify a novel panel of blood-based metabolites that are predictive, diagnostic, and/or prognostic of behavioral effects.

At 8 weeks of age, male rats were randomly assigned to groups and were gavage fed with full-spectrum cannabis extract (tetrahydrocannabinol/cannabidiol (THC/CBD) along with all other cannabis compounds, 15 mg/kg), broad-spectrum cannabis extract (CBD along with all other cannabis compounds, 15 mg/kg), or vehicle oil. Four hours after being gavage fed, behavioral assessments were determined using the open field test and the elevated plus maze. Following these assessments, serum was collected from all rats and the serum metabolites were identified and quantified by LC–MS/MS and 1H NMR spectroscopy.

We found that only rats treated with full-spectrum cannabis extract exhibited behavioral changes. Compared to vehicle-treated and broad-spectrum extract–treated rats, full-spectrum extract–treated rats demonstrated higher serum concentrations of the amino acid phenylalanine and long-chain acylcarnitines, as well as lower serum concentrations of butyric acid and lysophosphatidylcholines. This unique metabolomic fingerprint in response to cannabis extract administration is linked to behavioral effects and may represent a biomarker profile of cannabis-induced behavioral changes. If validated, this work may allow a metabolomics-based decision tree that would aid in the rapid diagnosis of cannabis-induced behavioral changes including cognitive impairment.

## Introduction

Cannabis has been used for centuries due to its medicinal benefits (Maayah et al., 2020b). Numerous existing full-spectrum cannabis extract products, that is, tetrahydrocannabinol/cannabidiol (THC/CBD), along with many other compounds such as terpenes and cannabinoids, have been used for the treatment of various conditions such as neuropathic pain and inflammation (Izzo et al., 2009; Maayah et al., 2020a; Maayah and Dyck, 2020). However, despite its efficacy in small patient studies (Maayah et al., 2020a; Maayah and Dyck, 2020), the clinical use of full-spectrum cannabis extract is limited due to some of the side effects such as behavioral changes (Misner and Sullivan, 1999; Maayah et al., 2020a; Maayah and Dyck, 2020). Behavioral side effects of full-spectrum cannabis extract involve impaired short-term memory and concentration (Misner and Sullivan, 1999; Maayah and Dyck, 2020) as well as delayed reaction time (Misner and Sullivan, 1999; Maayah and Dyck, 2020), that is, cognitive dysfunction (Misner and Sullivan, 1999; Maayah and Dyck, 2020). In addition, cannabis-related behavioral effects often involve reduced motor skills and coordination, which can eventually affect an individual’s overall quality of life (Misner and Sullivan, 1999; Maayah and Dyck, 2020) even for tasks like impairing the ability to drive (Hartman and Huestis, 2013; Lee et al., 2021). However, there is no evidence that the simple presence of cannabis impairs cognitive function or affects motor skills and coordination (Brubacher et al., 2019). Also, given that many cannabinoids are present in cannabis and a number of these can induce behavioral changes (Montone et al., 2020), a test designed to measure THC levels in biological fluid samples may be misleading as these levels may not be truly indicative of cognitive dysfunction or impairment in motor skills (Brubacher et al., 2019). Thus, there remains a crucial need to identify tests that can help in the detection of full-spectrum cannabis–related behavioral effects and then devise a real-time decision tree that would have an impact on different aspects of a user’s day-to-day activities like the ability to operate a motor vehicle.

To better understand what constitutes cannabis-induced behavioral effects, we have used quantitative metabolomics to identify a novel panel of blood-based metabolites that are predictive, diagnostic, and/or prognostic of cannabis-induced behavioral effects. If validated, this work may allow a simple and reliable decision tree that would aid in the rapid diagnosis of cannabis-induced behavioral effects including cognitive impairment.

## Methods

### Experimental Design and Treatment Protocol

All protocols involving rodents were approved by the University of Alberta Institutional Animal Care and Use Committee (Health Sciences) and conform to the Guide for the Care and Use of Laboratory Animals published by the United States National Institutes of Health (eighth edition; revised 2011). The University of Alberta adheres to the principles for biomedical research involving animals developed by the Council for International Organizations of Medical Sciences and complies with the Canadian Council on Animal Care guidelines.

Male Sprague–Dawley rats were purchased from Charles River Laboratories. All rats were housed under standard conditions (25°C, 12:12-h light/ dark cycle) with ad libitum access to food and water. At 8 weeks of age, rats were randomly assigned into groups and were gavage-fed with broad-spectrum cannabis extract (i.e., CBD extract along with all other compounds and cannabinoids, except THC) (15 mg/kg), full-spectrum cannabis extract products (i.e., THC/CBD extract along with all other compounds and cannabinoids) (15 mg/kg), or vehicle oil (Supplementary Table S1). Four hours after being gavage-fed, behavioral observation assessments and blood collection were performed. Blood samples were collected in evacuated blood collection tubes and then the samples were allowed to clot by leaving them undisturbed at room temperature for 15 min. Subsequently, the serum was separated by centrifugation of blood samples at 2,000 × g for 10 min at 4°C. Following centrifugation, the serum was transferred into clean polypropylene tubes and stored at -80°C.

### Behavioral Testing

Tests were performed during the light cycle by an experimenter blind to the group conditions. All testing apparatuses were cleaned with unscented soap and water and dried between each animal. The trainer was blinded to treatment groups and performed behavioral analysis also post session.

### Open Field Test

To assess general locomotor performance and exploratory activity, rats were placed in the center of an open-field arena (100 × 80 × 30 cm) for 5 min and video recorded from above (Walsh and Cummins, 1976). Offline video analysis of the distance traveled was performed using customized tracking software (https://github.com/cdoolin/rat-apps) (Walsh and Cummins, 1976). The total distance traveled, percentage of time spent, and distance traveled in the inner 45% of the arena were measured, as described previously (Walsh and Cummins, 1976).

### Elevated Plus Maze

To assess anxiety-like behavior, rats were placed in the junction of two open arms and two closed arms (each arm is 50 cm long and 10 cm wide) elevated 65 cm above the ground while being video recorded from above for 10 min (Itoh et al., 1990; Sharma and Kulkarni, 1992). Offline video analysis was performed using customized motion tracking software (https://github.com/cdoolin/rat-apps) to analyze the percentage of time spent in the open arms and total distance traveled (Itoh et al., 1990; Sharma and Kulkarni, 1992). Entries into the open and closed arms of the elevated plus maze (EPM) were counted when all 4 paws were located in the arm, as described previously (Itoh et al., 1990; Sharma and Kulkarni, 1992).

### Combined Direct Flow Injection and Liquid Chromatography–Tandem Mass Spectrometry Compound Identification and Quantification

We applied a targeted quantitative metabolomics approach to analyze the samples using a combination of direct injection mass spectrometry with a reverse-phase LC–MS/MS custom assay. This custom assay, in combination with an AB Sciex 4000 QTRAP (Applied Biosystems/MDS Sciex) mass spectrometer, can be used for the targeted identification and quantification of up to 143 different endogenous metabolites including amino acids, acylcarnitines, biogenic amines and derivatives, uremic toxins, glycerophospholipids, sphingolipids, and sugars (Sung et al., 2017;Foroutan et al., 2019; Foroutan et al., 2020). The method combines the derivatization and extraction of analytes and the selective mass spectrometric detection using multiple reaction monitoring (MRM) pairs. Isotope-labeled internal standards and other internal standards are used for metabolite quantification. The custom assay contains a 96-well deep well plate with a filter plate attached with sealing tape, and reagents and solvents used to prepare the plate assay. The first 14 wells were used for one blank, three zero samples, seven standards, and three quality control samples. For all metabolites, except organic acid, samples were thawed on ice and were vortexed and centrifuged at 13,000 ×g. Then 10 µl of each sample was loaded onto the center of the filter on the upper 96-well plate and dried in a stream of nitrogen. Subsequently, phenyl isothiocyanate was added for derivatization. After incubation, the filter spots were dried again using an evaporator. Extraction of the metabolites were then achieved by adding 300 µl of extraction solvent. The extracts were obtained by centrifugation into the lower 96-well deep well plate, followed by a dilution step with MS running solvent.

For organic acid analysis, 150 µl of ice-cold methanol and 10 µl of isotope-labeled internal standard mixture were added to 50 µl of the serum sample for overnight protein precipitation. Then it was centrifuged at 13,000× g for 20 min. After that, 50 µl of supernatant was loaded at the center of the wells of a 96-well deep well plate, followed by the addition of 3-nitrophenylhydrazine (NPH) reagent. After incubation for 2 h, a BHT stabilizer and water were added before LC–MS injection.

Mass spectrometric analysis was performed on an AB Sciex 4000 QTRAP^®^ tandem mass spectrometry instrument (Applied Biosystems/MDS Analytical Technologies, Foster City, CA) equipped with an Agilent 1260 series UHPLC system (Agilent Technologies, Palo Alto, CA). The samples were delivered to the mass spectrometer by an LC method, followed by a direct injection (DI) method. Data analysis was performed using Analyst 1.6.2.

### Nuclear Magnetic Resonance Spectroscopy

Serum samples contain a significant concentration of large–molecular weight proteins and lipoproteins, which affect the identification of the small–molecular weight metabolites by NMR spectroscopy. Therefore, a deproteinization step, involving ultrafiltration, as previously described (Psychogios et al., 2011), was therefore introduced in the protocol to remove serum proteins. Prior to filtration, 3-KDa cutoff centrifugal filter units (Amicon Microcon YM-3) were rinsed five times each with 0.5 ml of H_2_O and centrifuged (10,000 rpm for 10 min) to remove residual glycerol bound to the filter membranes. Aliquots of each serum sample were then transferred into the centrifuge filter devices and spun (10,000 rpm for 20 min) to remove macromolecules (primarily protein and lipoproteins) from the sample. The filtrates were checked visually for any evidence that the membrane was compromised, and for these samples, the filtration process was repeated with a different filter and the filtrate inspected again. The subsequent filtrates were collected, and the volumes were recorded. If the total volume of the sample was under 250 µl, an appropriate amount from a 150 mM KH_2_PO_4_ buffer (pH 7) was added until the total volume of the sample was 173.5 µ. Any sample that had to have buffer added to bring the solution volume to 173.5 µl was annotated with the dilution factor, and metabolite concentrations were corrected in the subsequent analysis. Subsequently, 46.5 µl of a standard buffer solution (54% D_2_O:46% 1.75 mM KH_2_PO_4_ pH 7.0 v/v containing 5.84 mM DSS (2,2-dimethyl-2-silcepentane-5-sulphonate), 5.84 mM 2-chloropyrimidine-5 carboxylate, and 0.1% NaN_3_ in H_2_O) was added to the sample.

The serum sample (250 µl) was then transferred to a 3-mm SampleJet NMR tube for subsequent spectral analysis. All 1H NMR spectra were collected on a 700 MHz Avance III (Bruker) spectrometer equipped with a 5-mm HCN Z-gradient pulsed-field gradient (PFG) cryoprobe. 1H NMR spectra were acquired at 25°C using the first transient of the NOESY pre-saturation pulse sequence (noesy1dpr), chosen for its high degree of quantitative accuracy (Saude et al., 2006). All FIDs (free induction decays) were zero-filled to 250K data points. The singlet produced by the DSS methyl groups was used as an internal standard for chemical shift referencing (set to 0 ppm), and for quantification, all 1H NMR spectra were processed and analyzed using an in-house version of the MAGMET automated analysis software package using a custom metabolite library. MAGMET allows for qualitative and quantitative analysis of an NMR spectrum by automatically fitting spectral signatures from an internal database to the spectrum. Each spectrum was further inspected by an NMR spectroscopist to minimize compound misidentification and misquantification. Typically, all visible peaks were assigned. Most of the visible peaks were annotated with a compound name. It has been previously shown that this fitting procedure provides absolute concentration accuracy of 90% or better (Zordoky et al., 2015; Ravanbakhsh et al., 2015).

### Cannabinoid Analysis Method

For cannabinoid analysis, 150 µl of ice-cold methanol and 10 µl of isotope-labeled internal standard mixture were added to 50 µl of each individual sample (PBS as blank sample, calibration standards, QC standards, and serum samples) for a 1-h protein precipitation. All the samples were then centrifuged at 13,000 × g for 20 min. For each sample, 180 µl of the supernatant was loaded into the center of corresponding wells of a 96-well deep well plate, followed by the addition of 90 µl of water to each well. The plate was then shaken at 600 rpm for 15 min before LC–MS/MS analysis. Mass spectrometric analysis was performed on an AB Sciex 5500 QTRAP^®^ tandem mass spectrometry instrument (Applied Biosystems/MDS Analytical Technologies, Foster City, CA) equipped with an Agilent 1290 series UHPLC system (Agilent Technologies, Palo Alto, CA). Data analysis was done using Analyst 1.6.3.

### Statistical Analysis

Results are shown as means ± SEM. Statistical analysis was carried out using GraphPad Prism software (version 7.04) (GraphPad Software, Inc., La Jolla, CA). The Shapiro–Wilk test was used to assess the normality of distribution of each parameter. One-way analysis of variance (ANOVA), followed by the Tukey–Kramer post hoc multiple comparison test, unpaired two-tailed *t*-test for normally distributed data, or Kruskal–Wallis test for non-normally distributed data were carried out to assess which treatment group(s) showed a significant difference.

For metabolomic data analysis, log-transformation was applied to all quantified metabolites to normalize the concentration distributions. Heat maps were generated with the concentrations of potential candidate metabolites, which were extracted with univariate analysis. It was generated without hierarchical cluster analysis unlike usual structures of heat maps and simply arranged by grouping similar metabolites together for use in pathway analysis through intuitive pattern discovery. The heat map displays an increase in each metabolite in relative concentration as red color and a decrease in a metabolite as blue color. The metabolites are listed at the left side of each row, and the subjects are shown at the bottom of each column. Partial least squares discriminant analysis (PLS-DA) score plots were used to compare serum metabolite data across and between study groups; 100-fold permutation tests were used to minimize the possibility that the observed separation of the PLS-DA was due to chance. Coefficient scores and least absolute shrinkage and selection operator (LASSO) algorithm were used to identify the most discriminating metabolites for group comparisons. A receiver operating characteristic (ROC) curve was determined. The ROC calculations included bootstrap 95% confidence intervals for the desired model specificity and other measures including accuracy and false discovery rates (FDR). Metabolite data analyses were done using MetaboAnalyst (Lopez-Hernandez et al., 2021).

## Results

### Full-Spectrum Cannabis Extract Induces Behavioral Changes in Rats

**FIGURE 1:**
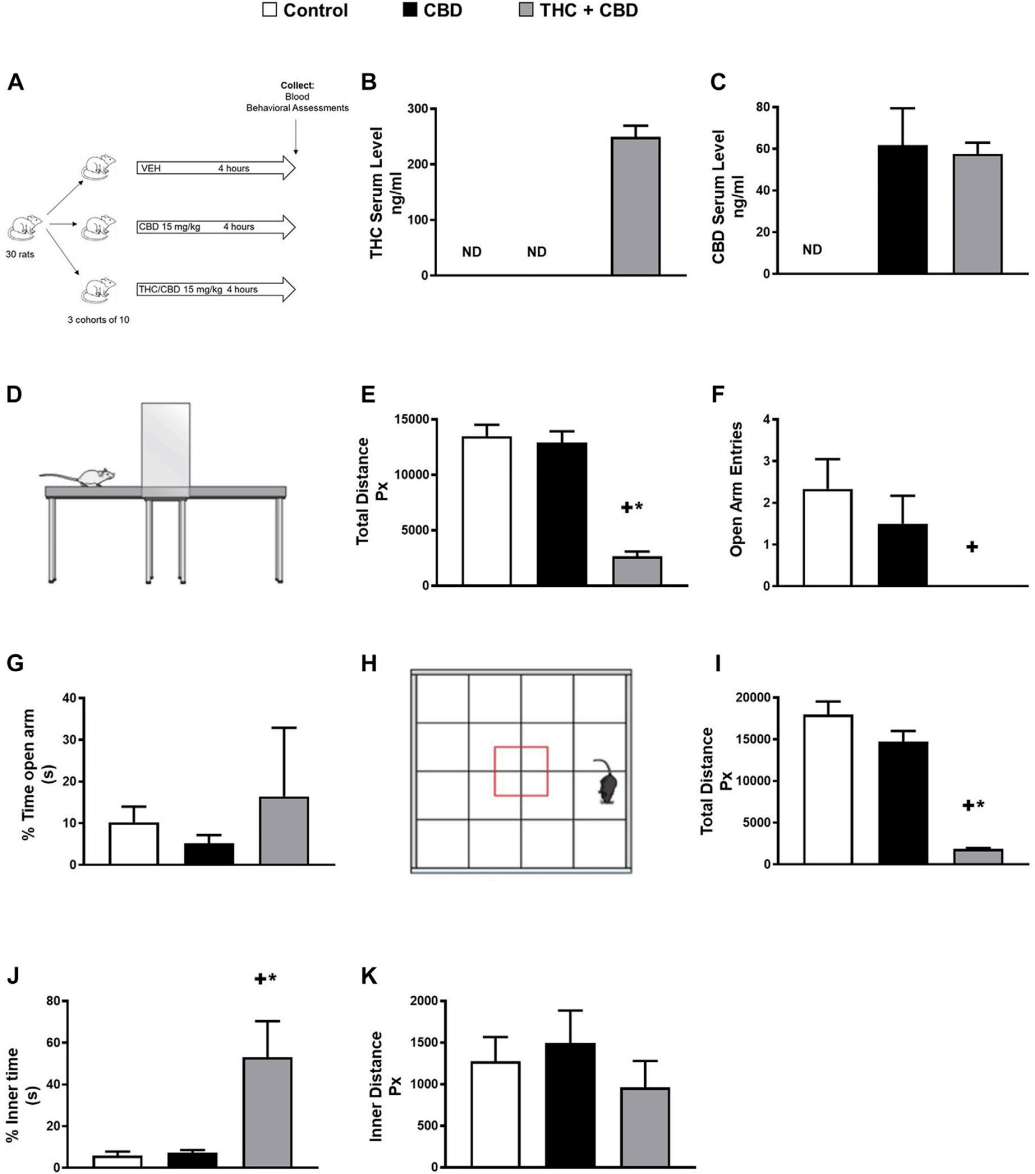
Full-spectrum cannabis extract induces behavioral changes in rats. **(A)** Scheme of study design for identifying novel metabolite biomarkers of behavioral changes in response to cannabis extracts. **(B)** THC serum concentration and **(C)** CBD serum concentration that was determined in vehicle-treated, broad-spectrum cannabis extract–treated, and full-spectrum cannabis extract–treated rats. **(D)** Image showing a rat in the elevated plus maze apparatus. **(E)** Total distance travelled, **(F)** open arm entries, and **(G)** % time open arm in vehicle-treated, broad-spectrum cannabis extract–treated and full-spectrum cannabis extract–treated rats (n = 6). **(H)** Image showing a rat in the open field apparatus, **(I)** total distance travelled, **(J)** % inner time, and **(K)** inner distance in vehicle-treated, broad-spectrum cannabis extract–treated, and full-spectrum cannabis extract–treated rats (n = 6). Results are shown as means ± SEM. Comparisons between three groups were made by one-way ANOVA with a Tukey–Kramer post hoc multiple comparison test or Kruskal–Wallis test. +*p*< 0.05 vs. vehicle control group. *p < 0.05 vs. broad-spectrum cannabis extract group. THC, tetrahydrocannabinol; CBD, cannabidiol.

Considering the fact that rats are commonly used animals in behavioral research (Feyissa et al., 2017), we sought to test the effect of full-spectrum and broad-spectrum cannabis extracts on behavioral changes in our rat model using a clinically relevant dose of cannabis (Huestis, 2007). Given that there is no evidence that the simple presence of cannabis (i.e., low concentration of THC) impairs behavior (Brubacher et al., 2019), the dosage of full-spectrum cannabis extract (15 mg/kg, per oral) we used in our rat model was selected from the literature from studies in rodents to provide the highest achievable concentration of THC in humans (Huestis, 2007). To do this, rats were treated with full-spectrum cannabis extract, broad-spectrum cannabis extract, or vehicle (Figures 1A–C). Interestingly, while broad-spectrum cannabis extract-treated rats did not demonstrate significant behavioral changes, rats treated with full-spectrum cannabis extract traveled significantly less distance and made significantly fewer open arm entries than broad-spectrum cannabis extract–treated and vehicle-treated rats in the elevated plus maze (Figures 1D–F). Taken together, these data suggest that rats administered with full-spectrum cannabis extract displayed a significant behavioral change.

To further assess general locomotor performance and exploratory activity, we performed open field tests in our rat model (Figure 1H). Our results show that full-spectrum cannabis extract–treated rats displayed a significant reduction in the total distance traveled in the open field (Figure 1I). In addition, full-spectrum cannabis extract–treated rats demonstrated a significant increase in the percentage of inner time compared to broad-spectrum cannabis extract–treated and vehicle-treated rats (Figure 1J). However, there was no difference between all experimental groups in the % time open arms in the elevated plus maze as well as the inner distance in the open field test (Figures 1G,K). Overall, our data indicate that full-spectrum cannabis extract induces clear behavioral effects in our rats.

### Serum Metabolite Profile Differences Between Full-Spectrum Cannabis Extract and Controls

**FIGURE 2:**
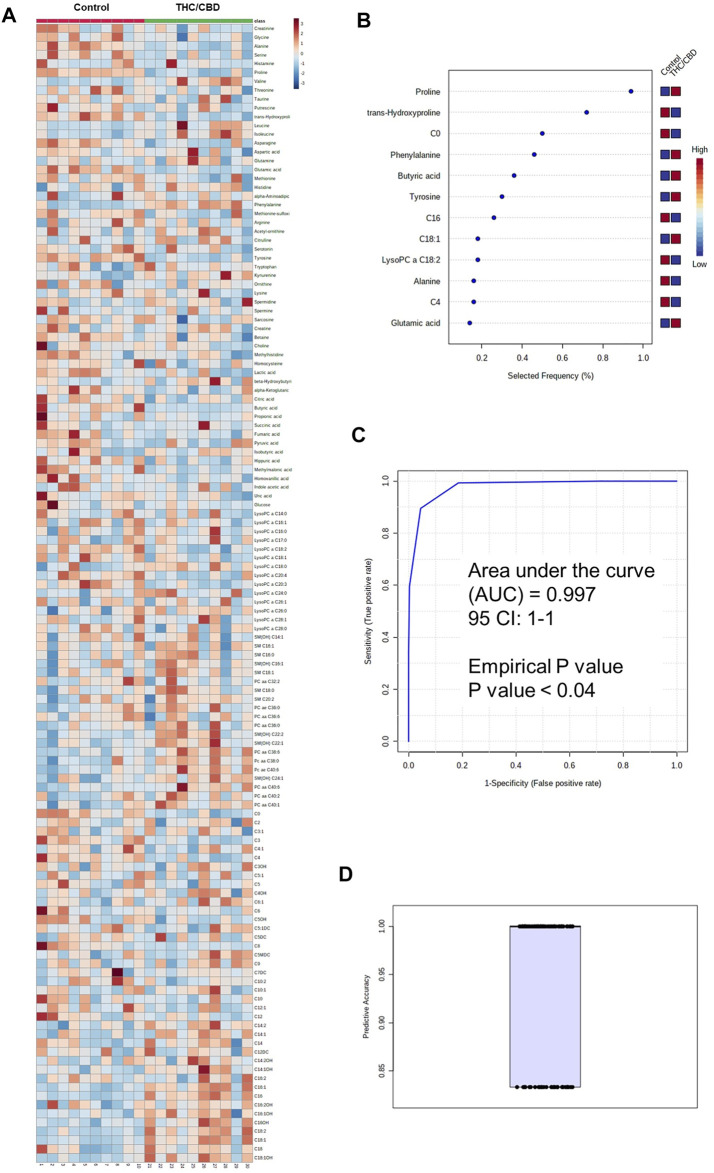
Metabolomic differences between full-spectrum cannabis extract and controls. **(A)** Heat map of metabolomic differences between full-spectrum cannabis extract and controls. Heat maps were generated with the concentrations of potential candidate metabolites with univariate analysis. Similar metabolites were arranged together for use in pathway analysis through intuitive pattern discovery. The heat map displays an increase in each metabolite in relative concentration as red color and a decrease in a metabolite as blue color. The metabolites are listed at the left side of each row, and the subjects are shown at the bottom of each column. **(B)** Rank of the different metabolites (the top 12) identified by the PLS-DA according to the selected frequency on the *x*-axis. The most discriminating metabolites are shown in descending order of their scores. The color boxes indicate whether metabolite concentration has increased (red) or decreased (blue) in vehicle-treated and full-spectrum cannabis extract–treated rats. **(C)** ROC curve of the metabolite model. **(D)** Predictive accuracy of the metabolite model in vehicle-treated and full-spectrum cannabis extract–treated rats. The figures were drawn via MetaboAnalyst software v 4.0 (https://www.metaboanalyst.ca).

Serum metabolomic analysis of a total of 181 analyzed metabolites from DI-MS (148 metabolites) and NMR (33 metabolites) revealed that the serum concentrations of some medium-chain and long-chain acylcarnitines, kynurenine, phosphatidylcholines (PC) such as PC ae C40:6, some lysophosphatidylcholines (LysoPC), sphingomyelins (SM) such as SM(OH) C22:2, and several amino acids such as phenylalanine were higher in full-spectrum cannabis extract–treated group than controls (Figure 2A, Supplementary Table S1). Conversely, the serum concentrations of some short-chain acylcarnitines such as C4, carnitine, LysoPC such as LysoPC a C18:2, LysoPC a C20:3 and LysoPC a C20:4, butyric acid, glutamic acid, methylmalonic acid, lactic acid, hippuric acid, homovanillic acid, alpha-ketoglutaric acid, uric acid, methionine sulfoxide, and several amino acids such as tyrosine were lower in full-spectrum cannabis extract–treated group than controls (Figure 2A, Supplementary Table S2).

To identify potential metabolite biomarkers of cannabis-related behavioral effects, PLS-DA was performed to find the most parsimonious model to discriminate full-spectrum cannabis extract–treated group from the controls. A small number of metabolites including phenylalanine, tyrosine, butyric acid, LysoPC a C18:2, C0, alanine, C4, C16, C18:1, proline, *trans*-hydroxyproline, and glutamic acid was able to discriminate full-spectrum cannabis extract–treated group from the controls. Receiver operating characteristic (ROC) curve analysis using only these selected metabolites produced an area under the curve (AUC) of 0.997 (Figure 2B). The permutation test’s result (*p*-value < 0.04) for model validation with an average accuracy of 0.948 indicated that the model was significant (Figures 2B,C). Intriguingly, since the aforementioned metabolite biomarkers are also detected in human and rodents with cognitive impairment (Ravaglia et al., 2004; Mapstone et al., 2014; Ashe et al., 2019; Heyck and Ibarra, 2019), our findings are highly suggestive that full-spectrum cannabis extract induces cognitive dysfunction in our rat model.

### Metabolomic Differences Between Full-Spectrum Cannabis Extract and Broad-Spectrum Cannabis Extract

**FIGURE 3:**
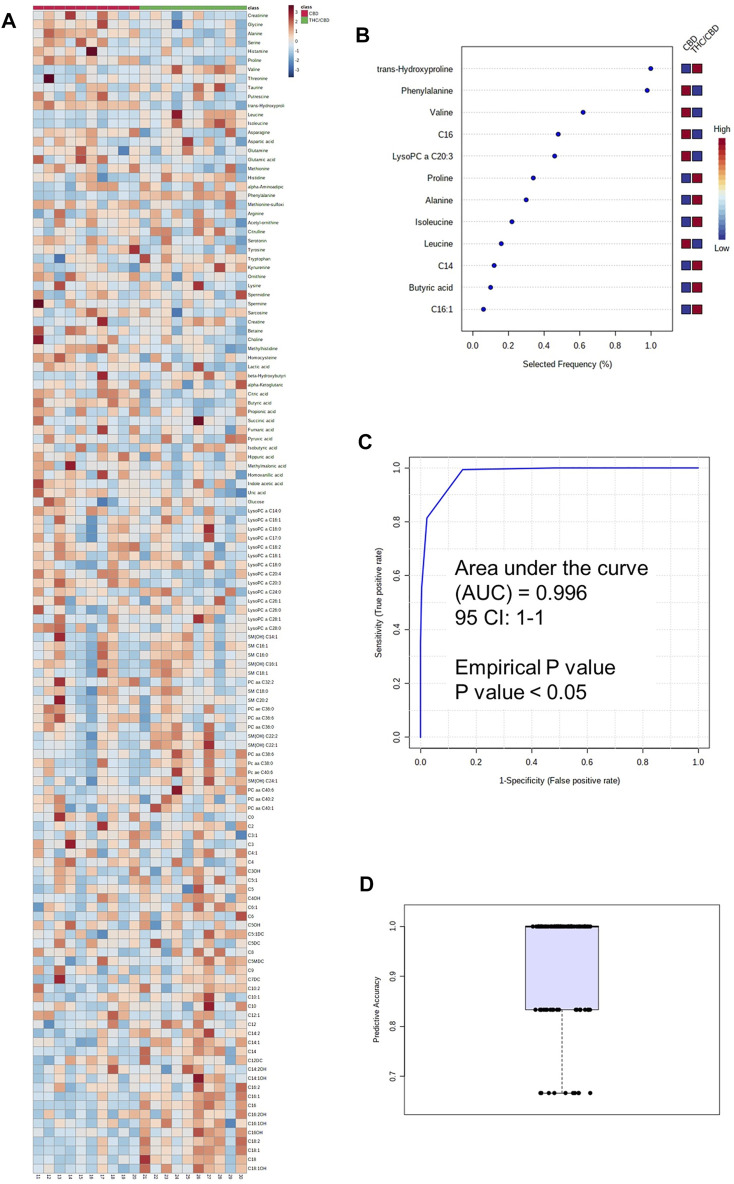
Metabolomic differences between full-spectrum cannabis extract and broad-spectrum cannabis extract. **(A)** Heat map of metabolomic differences between full-spectrum cannabis extract and broad-spectrum cannabis extract groups. Heat maps were generated with the concentrations of potential candidate metabolites with univariate analysis. Similar metabolites were arranged together for use in pathway analysis through intuitive pattern discovery. The heat map displays an increase in each metabolite in relative concentration as red color and a decrease in a metabolite as blue color. The metabolites are listed at the left side of each row, and the subjects are shown at the bottom of each column. **(B)** Rank of the different metabolites (the top 12) identified by the PLS-DA according to the selected frequency on the *x*-axis. The most discriminating metabolites are shown in descending order of their scores. The color boxes indicate whether metabolite concentration has increased (red) or decreased (blue) in broad-spectrum cannabis extract–treated and full-spectrum cannabis extract–treated rats. **(C)** ROC curve of the metabolite model. **(D)** Predictive accuracy of the metabolite model in broad-spectrum cannabis extract–treated and full-spectrum cannabis extract–treated rats. The figures were drawn via MetaboAnalyst software v 4.0 (https://www.metaboanalyst.ca).

The metabolomic analysis showed that the serum concentrations of some medium- and long-chain acylcarnitines, some amino acids such as phenylalanine, SM(OH) C24:1, SM(OH) C22:2, PC aa C40:6, tryptophan, and kynurenine were higher in the full-spectrum cannabis extract–treated group than the broad-spectrum cannabis extract–treated group, whereas the serum concentrations of C4, LysoPC a C20:3, LysoPC a C18:2, LysoPC a C20:4, LysoPC a C14:0, butyric acid, glutamic acid, hippuric acid, uric acid, methionine sulfoxide, methylhistidine, asparagine, histamine, creatinine, glycine, serotonin, and several amino acids were found to be lower in the full-spectrum cannabis extract–treated group than the broad-spectrum cannabis extract–treated group (Figure 3, Supplementary Table S3).

For the identification of a potential biomarker panel of metabolites, we performed a similar PLS-DA of metabolites from the full-spectrum cannabis extract–treated group and the broad-spectrum cannabis extract–treated group. Another small number of metabolites including phenylalanine, butyric acid, alanine, LysoPC a C20:3, C16:1, C14, C16, proline, *trans*-hydroxyproline, valine, isoleucine, and leucine discriminated full-spectrum cannabis extract–treated group from the broad-spectrum cannabis extract–treated rats. ROC curve analysis produced an AUC of 0.996 (Figure 3B). The permutation test’s result (*p*-value < 0.05) for model validation with an average accuracy of 0.932 indicated that the model was significant (Figures 3B,C). Based on our findings, it is clear that phenylalanine, butyric acid, alanine, C16, proline, and *trans*-hydroxyproline discriminated the full-spectrum cannabis extract–treated group from the both control and broad-spectrum cannabis extract–treated rats (Figure 2B, 3B).

### Full-Spectrum Cannabis Extract Significantly Reduces Phenylalanine Hydroxylase Enzyme Activity

**FIGURE 4:**
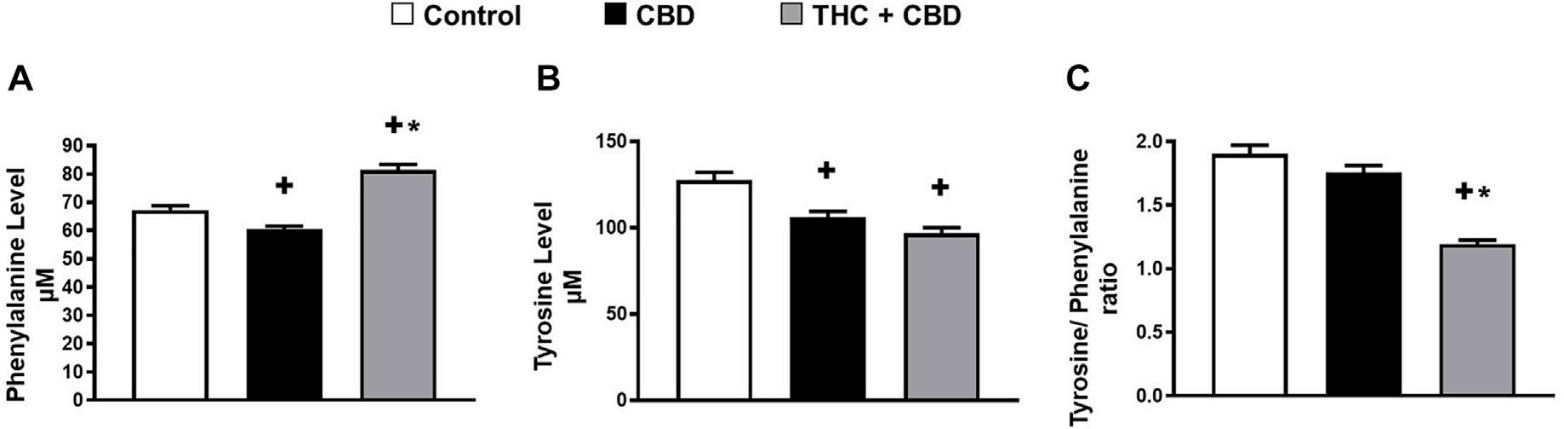
Full-spectrum cannabis extract may significantly reduce phenylalanine hydroxylase enzyme activity. **(A)** Serum level of phenylalanine; **(B)** serum level of tyrosine, and **(C)** the ratio of tyrosine to phenylalanine (phenylalanine hydroxylase) in vehicle-treated, broad-spectrum cannabis extract–treated, and full-spectrum cannabis extract–treated rats (n = 10). Results are shown as mean ± SEM. Comparisons between three groups were made by one-way ANOVA with a Tukey–Kramer post hoc multiple comparison test. +*p*< 0.05 vs. vehicle group. *p < 0.05 vs. full-spectrum cannabis extract group.

Given that 1) phenylalanine discriminated full-spectrum cannabis extract group from both control and broad-spectrum cannabis extract-treated rats (Figure 2B, 3B), and 2) a reduction in the activity of phenylalanine hydroxylase enzyme is known to cause behavioral problems and cognitive dysfunction (Ravaglia et al., 2004; Ashe et al., 2019), we sought to test the effect of full-spectrum cannabis extract on phenylalanine hydroxylase enzyme activity by measuring the ratio of tyrosine/phenylalanine. Interestingly, we found that the full-spectrum cannabis extract–treated group displayed a significant reduction in the ratio of tyrosine/phenylalanine compared to both control and broad-spectrum cannabis extract–treated groups, suggesting that the behavioral effect induced by the full-spectrum cannabis extract might be attributed, at least in part, to a reduction in the activity of phenylalanine hydroxylase enzyme (Figure 4).

## Discussion

Herein, we tested the effect of cannabis extracts on behavioral changes in our rat model using a clinically relevant dose of cannabis (Huestis, 2007). Given that there is no evidence that the simple presence of cannabis (i.e., low concentration of THC) impairs behavior (Brubacher et al., 2019), the dosage of the full-spectrum cannabis extract we used in our rat model was selected from studies in rodents to provide the highest achievable concentration of THC in humans (Huestis, 2007). Interestingly, while the broad-spectrum cannabis extract administered in this study did not result in any signs of behavioral changes, the full-spectrum cannabis extract displayed significant behavioral changes in our rat model. Thus, we conclude that consistent with other studies (Misner and Sullivan, 1999; Maayah and Dyck, 2020), THC is the primary component of cannabis that is responsible for behavioral changes (Misner and Sullivan, 1999). Notably, our observations are also in agreement with studies showing that only high concentration of THC induces behavioral changes (Brubacher et al., 2019; Lee et al., 2021), suggesting that the behavioral effect of the full-spectrum cannabis extract is dose-dependent and supports the notion that the consumption of low dose of the full-spectrum cannabis extract is not associated with significant behavioral changes (Brubacher et al., 2019).

To better understand what constitutes the cannabis-induced behavioral effect, we used quantitative metabolomics to identify a novel panel of blood-based metabolites that are predictive, diagnostic, and/or prognostic of cannabis behavioral effect. If validated, this could be used as a test to help in the reliable detection of full-spectrum cannabis extract–related behavioral changes. For instance, in the present study, we show that phenylalanine hydroxylase is implicated in the behavioral changes of the full-spectrum cannabis extract. Our findings support the hypothesis that an impaired conversion of the reduced amino acid phenylalanine to tyrosine as a consequence of the phenylalanine hydroxylase activity may contribute to the behavioral changes induced by the full-spectrum cannabis extract (Ashe et al., 2019). In agreement with this hypothesis, elevated phenylalanine concentrations in blood were found in patients with mild cognitive impairment and are known to cause severe intellectual disability and cognitive impairment (Ravaglia et al., 2004; Ashe et al., 2019). Our observations are also in agreement with several reports showing that impairment in phenylalanine hydroxylase may be linked to the change in the production of other catecholamine neurotransmitters such as dopamine, epinephrine, and norepinephrine (Romani et al., 2017; Winn et al., 2018). Thus, it is likely that phenylalanine hydroxylase activity and/or phenylalanine concentrations in the blood could be used as a diagnostic and prognostic tool of cannabis-induced behavioral effect including cognitive dysfunction.

The present study also sheds light on the potential contribution of microbial by-products, in particular butyric acid, to full-spectrum cannabis extract–induced behavioral changes. This finding is congruent with the concept that the gut microbiome plays a vital role in mental health and neurological conditions such as motor impairment and cognitive dysfunction (Tooley, 2020). Given the fact that 1) butyric acid is known to improve cognitive function and motor skills (Cantu-Jungles et al., 2019; Heyck and Ibarra, 2019), and 2) we found that the full-spectrum cannabis extract dramatically reduces the serum concentration of butyric acid, we speculate that the behavioral changes in our rat model is also attributed to, at least in part, the disturbances in gut microbiome composition and a subsequent reduction in butyric acid. Based on this, in addition to the utilization of the serum level of butyric acid as a diagnostic tool of the cannabis behavioral effect, we suggest that butyrogenic prebiotic fibers or sodium butyrate could be attempted in preclinical and clinical studies to lessen cannabis-induced behavioral changes including cognitive dysfunction. Thus, butyrogenic prebiotic fibers or sodium butyrate may hold promise as a repurposed therapy to reduce cannabis-related behavioral effects.

Numerous other metabolic perturbations were detected in our full-spectrum cannabis extract rat model that warrants discussion. For instance, the serum concentrations of long-chain acyl carnitine are significantly higher in full-spectrum cannabis extract–treated rats than broad-spectrum cannabis extract and controls. Given the fact that carnitine and its acyl derivatives play a vital role in mitochondrial metabolism and fatty acid uptake, the higher long-chain acyl carnitine concentration in full-spectrum cannabis extract–treated rats may suggest inefficient whole-body β-oxidation (Adams et al., 2009). In addition to inefficient β-oxidation and the disruption in mitochondrial energy production (Wajner and Amaral, 2015), long-chain acyl carnitine derivatives are themselves pro-inflammatory, neurotoxic, and involved in the pathophysiology of the cerebral damage (Rutkowsky et al., 2014; Wajner and Amaral, 2015). Thus, we assume that long-chain acyl carnitine derivatives may contribute, at least in part, to the full-spectrum cannabis extract–induced behavioral effect in our rat model. In contrast to long-chain acyl carnitine, serum concentrations of neuroprotective LysoPCs, in particular LysoPC a C18:2, were lower in full-spectrum cannabis extract–treated rats than in broad-spectrum cannabis extract–treated and control rats (Semba, 2020). Interestingly, our observations are in agreement with those of an important recent study on patients with mild cognitive impairment and Alzheimer’s disease where LysoPC a C18:2 was found to be lower in patients with mild cognitive impairment (Mapstone et al., 2014). Thus, it is likely that LysoPC a C18:2 concentrations in blood could be used as a diagnostic and prognostic tool of cannabis-induced cognitive impairment.

Since we have detected distinct metabolomic fingerprints of the full-spectrum cannabis extract–treated rat model, we sought to use these fingerprints to discover novel panels of biomarkers that are predictive, diagnostic, and/or prognostic of the cannabis-behavioral effect, which can be used later as a test to help in the rapid detection of full-spectrum cannabis–related behavioral changes including cognitive dysfunction and impaired motor skills. Using phenylalanine, butyric acid, alanine, C16, proline, and *trans*-hydroxyproline, we discovered a novel panel of biomarkers that reliably distinguishes the full-spectrum cannabis extract from the broad-spectrum cannabis extract and control in rats. Thus, our newly identified panel of metabolites may have potential to be used clinically in the diagnosis of a cannabis-related behavioral effect and then utilized to devise a real-time decision tree that allows people to work and operate a motor vehicle. To the best of our knowledge, this is the first report to identify a set of biomarkers that can be used as novel metabolite biomarkers of the behavioral effect in response to the consumption of cannabis extracts.

In summary, our results suggest that decreased phenylalanine hydroxylase activity, low serum level of butyric acid and LysoPC a C18:2, and increased long-chain acyl carnitine may play a crucial role in the full-spectrum cannabis extract–induced behavioral effects in our rat model. We also used an unbiased and systematic approach to identify novel metabolite biomarkers of behavioral effect in response to the full-spectrum cannabis extract. Thus, once our findings are clinically validated and refined, our work may allow a simple decision tree that would aid in the rapid diagnosis of cannabis-related behavioral effects. This would have potential applicability in different aspects of a users’ day-to-day activities such as operating a motor vehicle or heavy machinery.

## Data Availability

The original contributions presented in the study are included in the article/Supplementary Material, further inquiries can be directed to the corresponding author.
