# Supplementary material for: Metabolomic Fingerprint of Behavioral Changes in Response to Full-Spectrum Cannabis Extracts
Source: Front Pharmacol. 2022 Jan 25;13:831052. doi: 10.3389/fphar.2022.831052 (PMC8822156; doi:10.3389/fphar.2022.831052)
Supplement: Supplementary file 1 [file Table1.DOCX]

**S1Table. List of cannabinoids and terpenes in our Cannabis extract**

| **Name** | **%** |
| --- | --- |
|  |  |
| Cannabinol | <0.05 |
| Cannabidiolic acid | <0.05 |
| D9-Tetrahydrocannabinolic acid | <0.05 |
| alpha-Bisabolol | 0.019 |
| alpha-Cedrene | <0.001 |
| alpha-Humulene | 0.007 |
| alpha-Pinene | 0.002 |
| alpha-Terpinene | <0.001 |
| alpha-Terpineol | 0.006 |
| beta-Myrcene | 0.004 |
| beta-Ocimene | <0.001 |
| beta-Pinene | <0.001 |
| Borneol isomers | 0.007 |
| Camphene | <0.001 |
| Camphor isomers | <0.001 |
| Caryophyllene oxide | <0.001 |
| Cedrol | <0.001 |
| cis-Nerolidol | <0.001 |
| Eucalyptol | <0.001 |
| Fenchone isomers | 0.004 |
| Fenchyl alcohol | 0.004 |
| gamma-Terpinene | 0.003 |
| gamma-Terpineol | <0.001 |
| Geraniol | <0.001 |
| Geranyl acetate | <0.001 |
| Guaiol | 0.0012 |
| Hexa-hydrothymol | <0.001 |
| Isoborneol | <0.001 |
| Isopulegol | <0.001 |
| Limonene | 0.003 |
| Linalool | <0.001 |
| Nerol | <0.001 |
| p-Mentha-1,5-diene | <0.001 |
| Pulegone | <0.001 |
| Sabinene | <0.001 |
| Sabinene hydrate | <0.001 |
| Terpinolene | 0.004 |
| trans-beta-Ocimene | <0.001 |
| trans-Caryophyllene | 0.012 |
| trans-Nerolidol | <0.001 |
| Valencene | <0.001 |
| 3-Carene | <0.001 |
