# Supplementary material for: Metabolomic Fingerprint of Behavioral Changes in Response to Full-Spectrum Cannabis Extracts
Source: Front Pharmacol. 2022 Jan 25;13:831052. doi: 10.3389/fphar.2022.831052 (PMC8822156; doi:10.3389/fphar.2022.831052)
Supplement: Supplementary file 2 [file Table2.DOCX]

**S2Table. Univariate Analysis of THC vs. Control.**

| **Metabolite** | **p-value** | **Mean (SD)** | | **THC / Control** | **Fold Change** |
| --- | --- | --- | --- | --- | --- |
|  |  | **THC** | **Control** |  |  |
| Number of rats | - | 10 | 10 | - | - |
| Proline | 1.86E-06 | 160.4 (25.9) | 228 (14.4) | Down | 0.703 |
| trans-Hydroxyproline | 1.22E-05 | 42 (3.8) | 54 (5.4) | Down | 0.77 |
| C0 | 2.81E-05 | 25 (2.9) | 33 3.6) | Down | 0.75 |
| Tyrosine | 3.29E-05 | 96 (5.9) | 127 (8.01) | Down | 0.75 |
| Phenylalanine | 3.49E-05 | 67 (6.6) | 81 (4.9) | Up | 1.2 |
| Alanine | 4.32E-05 | 430 (66.1) | 626 (104.6) | Down | 0.68 |
| C16 | 5.28E-05 | 0.16 (0.04) | 0.09 (0.01) | Up | 1.7 |
| Butyric acid | 6.58E-05 | 1.6 (1.04) | 7.8 (5.1) | Down | 0.21 |
| C18:2 | 0.000184 | 0.43 (.013) | 0.23 (.006) | Up | 1.8 |
| LysoPC a C18:2 | 0.000202 | 42.6 (8.6) | 60.6 (7.8) | Down | 0.7 |
| Glutamic acid | 0.000257 | 64.6 (3.9) | 80.1 (10.2) | Down | 0.8 |
| C4 | 0.000312 | 0.34 (0.09) | 0.54 (0.11) | Down | 0.64 |
| C3 | 0.000451 | 0.36 (0.05) | 0.54 (0.11) | Down | 0.68 |
| C18:1 | 0.000471 | 0.14 (0.045) | 0.07 (0.016) | Up | 1.9 |
| C16:1 | 0.000517 | 0.039 (0.007) | 0.028 (0.004) | Up | 1.4 |
| C18:1OH | 0.000633 | 0.017 (0.0025) | 0.025 (0.005) | Up | 1.44 |
| LysoPC a C20:3 | 0.000754 | 3.9 (0.4) | 5 (0.75) | Down | 0.78 |
| Asparagine | 0.000897 | 70.5 (14) | 93.4 (13) | Down | 0.75 |
| C16OH | 0.001399 | 0.025 (0.005) | 0.019 (0.002) | Up | 1.36 |
| Valine | 0.002106 | 245 (34.5) | 204 (15.1) | Up | 1.2 |
| Isoleucine | 0.002729 | 100.4 (13.02) | 85.1 (6.1) | Up | 1.18 |
| Methylmalonic acid | 0.003335 | 0.24 (0.09) | 0.48 (0.22) | Down | 0.5 |
| Leucine | 0.003509 | 208.4 (53.7) | 155.4 (15.6) | Up | 1.34 |
| C8 | 0.003672 | 0.014 (0.002) | 0.027 (0.014) | Down | 0.52 |
| Lactic acid | 0.003780 | 1623 (772) | 2561 (607) | Down | 0.64 |
| C14:1OH | 0.004213 | 0.023 (0.004) | 0.018 (0.002) | Up | 1.25 |
| C14:2 | 0.005273 | 0.017 (0.003) | 0.013 (0.002) | Up | 1.3 |
| SM(OH) C22:1 | 0.006004 | 10.9 (1.6) | 9 (1.2) | Up | 1.2 |
| SM(OH) C22:2 | 0.007139 | 3.8 (0.5) | 3.18 (0.3) | Up | 1.2 |
| Methionine-sulfoxide | 0.007401 | 5.2 (2.1) | 7.3 (1.4) | Down | 0.7 |
| Pc ae C40:6 | 0.007476 | 2.3 (0.4) | 1.8 (0.23) | Up | 1.23 |
| PC aa C40:6 | 0.007666 | 37.8 (10.9) | 27.2 (4.8) | Up | 1.38 |
| SM(OH) C24:1 | 0.008966 | 2.87 (0.3) | 2.39 (0.4) | Up | 1.2 |
| PC aa C38:6 | 0.009204 | 50.5 (12) | 37.9 (5.5) | Up | 1.33 |
| LysoPC a C20:4 | 0.009277 | 26.2 (3.5) | 31.1 (4.1) | Down | 0.84 |
| PC aa C40:1 | 0.011514 | 0.3 (0.02) | 0.27 (0.03) | Up | 1.14 |
| C14:1 | 0.011833 | 0.058 (0.009) | 0.047 (0.009) | Up | 1.25 |
| Pc aa C38:0 | 0.013504 | 1.39 (0.15) | 1.18 (0.18) | Up | 1.17 |
| Serine | 0.014039 | 237.4 (24.5) | 269 (29) | Down | 0.88 |
| Hippuric acid | 0.015952 | 4.5 (1.7) | 6.7 (1.9) | Down | 0.67 |
| Homovanillic acid | 0.021309 | 0.17 (0.003) | 0.02 (0.006) | Down | 0.76 |
| Kynurenine | 0.02374 | 4.5 (0.7) | 3.8 (0.5) | Up | 1.18 |
| alpha-Ketoglutaric acid | 0.025938 | 10.2 (3.1) | 13.4 (3) | Down | 0.76 |
| Uric acid | 0.02937 | 46.8 (17) | 68.1 (23) | Down | 0.68 |
| Fumaric acid | 0.030151 | 1.7 (0.27) | 2.23 (0.5) | Down | 0.78 |
| C5OH | 0.032402 | 0.027 (0.004) | 0.033 (0.006) | Down | 0.82 |
| LysoPC a C18:1 | 0.032786 | 26.7 (3) | 30.4 (3.9) | Down | 0.88 |
| LysoPC a C18:0 | 0.033929 | 111 (8.6) | 100.5 (11.7) | Up | 1.1 |
| Glycine | 0.035602 | 321 (44.8) | 368 (44.9) | Down | 0.87 |
