# Supplementary material for: Metabolomic Fingerprint of Behavioral Changes in Response to Full-Spectrum Cannabis Extracts
Source: Front Pharmacol. 2022 Jan 25;13:831052. doi: 10.3389/fphar.2022.831052 (PMC8822156; doi:10.3389/fphar.2022.831052)
Supplement: Supplementary file 3 [file Table3.DOCX]

**S3Table. Univariate Analysis of THC/CBD vs. CBD.**

| **Metabolite** | **p-value** | **Mean (SD)** | | **(THC/CBD) / CBD** | **Fold Change** |
| --- | --- | --- | --- | --- | --- |
|  |  | **THC/CBD** | **CBD** |  |  |
| Number of rats | - | 10 | 10 | - | - |
| Phenylalanine | 3.32E-08 | 81.3 (6.6) | 60.4 (3.5) | Up | 1.35 |
| trans-Hydroxyproline | 8.32E-08 | 42.05 (3.8) | 55.9 (2.9) | Down | 0.75 |
| Valine | 1.43E-05 | 245 (34) | 179 (16) | Up | 1.36 |
| LysoPC a C20:3 | 1.58E-05 | 3.9 (0.4) | 5.19 (0.55) | Down | 0.75 |
| C16 | 2.58E-05 | 0.169 (0.04) | 0.09 (0.01) | Up | 1.8 |
| Alanine | 2.86E-05 | 430 (66) | 609 (79) | Down | 0.89 |
| Proline | 3.85E-05 | 160.4 (25) | 218 (22) | Down | 0.73 |
| Isoleucine | 4.72E-05 | 100.5 (13) | 78 (5.4) | Up | 1.28 |
| Leucine | 0.00015153 | 208 (53) | 138.6 (13) | Up | 1.5 |
| Butyric acid | 0.00023304 | 1.6 (1.04) | 3.85 (0.9) | Down | 0.42 |
| LysoPC a C18:2 | 0.00082466 | 42.6 (8.6) | 59 (9.4) | Down | 0.71 |
| C16:1 | 0.00084831 | 0.039 (0.007) | 0.028 (0.004) | Up | 1.39 |
| C18:2 | 0.0012295 | 0.043 (0.013) | 0.025 (0.007) | Up | 1.6 |
| C18:1 | 0.0017756 | 0.14 (0.045) | 0.077 (0.02) | Up | 1.8 |
| LysoPC a C20:4 | 0.0023344 | 26.2 (3.5) | 31.1 (4.3) | Down | 0.8 |
| Methylhistidine | 0.0035906 | 8.5 (1.07) | 10.3 (1.26) | Down | 0.82 |
| C14 | 0.0036823 | 0.06 (0.013) | 0.044 (0.006) | Up | 1.38 |
| Glutamic acid | 0.004442 | 64.6 (3.9) | 79.5 (14.3) | Down | 0.81 |
| C16OH | 0.0044812 | 0.025 (0.005) | 0.019 (0.0028) | Up | 1.32 |
| Tryptophan | 0.005477 | 116.9 (9.9) | 101.74 (11) | Up | 1.14 |
| C18 | 0.0059509 | 0.067 (0.015) | 0.05 (0.006) | Up | 1.32 |
| C18:1OH | 0.0059885 | 0.025 (0.005) | 0.0188 (0.002) | Up | 1.33 |
| Asparagine | 0.0060296 | 70.5 (14) | 85 (7.5) | Down | 0.82 |
| SM(OH) C22:1 | 0.0067679 | 10.9 (1.6) | 9.11 (1.07) | Up | 1.19 |
| SM(OH) C24:1 | 0.0097971 | 2.87 (0.31) | 2.46 (0.31) | Up | 1.16 |
| PC aa C32:2 | 0.010266 | 0.77 (0.17) | 1.04 (0.26) | Down | 0.74 |
| Kynurenine | 0.010618 | 4.5 (0.7) | 3.58 (0.8) | Up | 1.26 |
| C14:1 | 0.014001 | 0.058 (0.009) | 0.047 (0.008) | Up | 1.23 |
| Methionine-sulfoxide | 0.015665 | 5.2 (2.1) | 7.5 (1.8) | Down | 0.67 |
| Citric acid | 0.01856 | 153 (12) | 170 (15.8) | Down | 0.9 |
| C14:2 | 0.019255 | 0.0174 (0.0029) | 0.0144 (0.002) | Up | 1.2 |
| Histamine | 0.022105 | 0.33 (0.45) | 0.97 (0.44) | Down | 0.34 |
| PC aa C40:6 | 0.02433 | 37.8 (10.9) | 28.9 (5.2) | Up | 1.3 |
| Uric acid | 0.024454 | 46.8 (17.6) | 64.11 (11.6) | Down | 0.73 |
| Glycine | 0.031885 | 321 (44) | 365 (38) | Down | 0.87 |
| Creatinine | 0.03281 | 17.9 (1.3) | 19.5 (1.8) | Down | 0.91 |
| C14:1OH | 0.033154 | 0.023 (0.004) | 0.02 (0.0025) | Up | 1.17 |
| LysoPC a C14:0 | 0.033289 | 9.3 (4) | 13 (3.4) | Down | 0.72 |
| C6:1 | 0.03505 | 0.016 (0.0027) | 0.013 (0.002) | Up | 1.2 |
| C4 | 0.03945 | 0.34 (0.09) | 0.42 (0.077) | Down | 0.8 |
| Serotonin | 0.041265 | 5.9 (4) | 9.4 (3) | Down | 0.6 |
| Serine | 0.044287 | 237 (24) | 263.9 (30) | Down | 0.89 |
| Betaine | 0.044497 | 151 (28) | 189.5 (45) | Down | 0.79 |
| SM(OH) C22:2 | 0.046245 | 3.8 (0.58) | 3.3 (0.45) | Up | 1.15 |
| Hippuric acid | 0.048455 | 4.5 (1.7) | 5.8 (1.2) | Down | 0.76 |
